# Insights into kinetics, thermodynamics, and mechanisms of chemically activated sunflower stem biochar for removal of phenol and bisphenol-A from wastewater

**DOI:** 10.1038/s41598-024-54907-y

**Published:** 2024-02-21

**Authors:** Lakshmi Prasanna Lingamdinne, Ganesh Kumar Reddy Angaru, Chandrika Ashwinikumar Pal, Janardhan Reddy Koduru, Rama Rao Karri, Nabisab Mujawar Mubarak, Yoon-Young Chang

**Affiliations:** 1 Department of Environmental Engineering, Kwangwoon University, Seoul, 01897 Republic of Korea; 2 Present Address: Petroleum and Chemical Engineering, Faculty of Engineering, Universiti Teknologi Brunei, Bandar Seri Begawan, BE1410 Brunei; 3 Department of Chemistry, School of Chemical Engineering and Physical Sciences, Lovely Professional University, Jalandhar, Punjab India

**Keywords:** Sunflower stem, Activated carbon, Phenol, Bisphenol A, Adsorption, Environmental sciences, Materials science

## Abstract

This study synthesized a highly efficient KOH-treated sunflower stem activated carbon (KOH-SSAC) using a two-step pyrolysis process and chemical activation using KOH. The resulting material exhibited exceptional properties, such as a high specific surface area (452 m^2^/g) and excellent adsorption capacities for phenol (333.03 mg/g) and bisphenol A (BPA) (365.81 mg/g). The adsorption process was spontaneous and exothermic, benefiting from the synergistic effects of hydrogen bonding, electrostatic attraction, and stacking interactions. Comparative analysis also showed that KOH-SSAC performed approximately twice as well as sunflower stem biochar (SSB), indicating its potential for water treatment and pollutant removal applications. The study suggests the exploration of optimization strategies to further enhance the efficiency of KOH-SSAC in large-scale scenarios. These findings contribute to the development of improved materials for efficient water treatment and pollution control.

## Introduction

Removal of phenolic compounds, including phenol and its derivatives, which are ubiquitous pollutants created during the production of many industries, including paper, medicine, metals, plastics, pesticides, and resins, is one of the most serious issues in the field of water treatment^1^. Similarly, plastics manufacturing businesses use a lot of bisphenol-A (BPA), a phenolic endocrine-disrupting chemical (EDC), as a monomer to make polycarbonate and epoxy resin. EDCs include industrial products like BPA, nonylphenol, several other phenol group chemicals, and organic substances containing one or more phenolic group(s), such as estrogens^2^. Because of their physicochemical characteristics and chemical bond configurations, these chemicals have the potential to cause harm to aquatic life, alter endocrine systems, and be hazardous to human health^3^. They can disrupt the reproductive systems of both humans and animals, raise the risk of testicular, breast, and ovarian cancer, and reduce fertility by mimicking or blocking the action of natural hormones^4^. BPA is found in many water sources, seeps into the water during manufacture, and leaches from wastes containing single-used polymers^5^. The main sources of phenol and BPA released into water systems are probably the discharge of industrial wastewater and municipal effluent.

Therefore, developing effective, user-friendly, and cost-effective technology to remove BPA and phenol from water is crucial. Thus far, several strategies have been proposed in earlier studies for removing BPA and phenol, including microbial degradation, membrane filtration, advanced oxidation, electrochemical techniques, and adsorption^6–10^. Adsorption has been found to be better since it is less costly, easier to plan and implement, and less likely to result in unintentionally creating additional dangerous chemicals^10^. An economical and efficient adsorbent must be used to ensure efficient wastewater treatment. Agricultural leftovers and garbage are often inexpensive, readily available, environmentally beneficial, and nearly non-toxic^11^. Using or converting agricultural waste into added-value products is another idea based on the "green chemistry" theory. In this case, various agricultural wastes are utilized as starting points for the production of adsorbents, such as activated carbon and biochar, that may be used to purify water. Corn cobs^12^, coconut shells^13^, rice straw^14^, plum stones^15^, tea waste^16^, and agricultural wastes^17^ are a few examples.

Sunflowers, or *Helianthus annuus*, are a major oilseed crop grown worldwide to provide exceptional oil and dietary fiber that is very beneficial to humans^18^. Among the top producers of sunflower seed oil are Turkey, Argentina, Ukraine, and Russia^19^. There is an increasing need for nutritional sunflower seeds, oil, and by-products due to the global population increase^20^. Sunflower leftovers, including stems, leaves, and heads, are produced in vast quantities and left in the fields after the seed is harvested. Often, it is a serious environmental risk to dispose of this trash from sunflowers in an economical way^21^.

Furthermore, due to its lignin-based composition and many oxygen-containing functionalities, such as hydroxyl and carboxyl moieties, sunflower biomass exhibits extensive chemical diversity and its vast accessibility^22,23^. In this context, it may be best to use sunflower stems as a raw material to produce activated carbon. Because of its physicochemical qualities, which include large surface area, natural availability, robust micropore pores, exceptional adsorption capacity, and structural properties, biochar has been employed by several studies to remove organic contaminants^24,25^. Importantly, biochar has a high concentration of oxygen-containing groups, such as –C–O, –COOH, O–C–O, and –OH, which might be important active sites for the interaction of contaminants^26^.

Unfortunately, the insufficiencies in biochar's large surface area and adsorption efficiency have hindered its practical use^11^. However, the biochar's porosity, which results only from pyrolysis, is minuscule. The pore structure of biochar must thus be customized. It has been shown that activation is the best method for increasing the porosity of the biochar. Biochar's porosity is heavily impacted by the activator and activation technique^27,28^. When it comes to activators, KOH is generally the most advantageous since it has a low activation temperature, produces higher yields, and has better-developed microporosity with an exceptionally high surface area among other activators like ZnCl_2_^31^, K_2_CO_3_^30^, and H_3_PO_4_^29^, among others^32,33^. Over 700 °C, KOH vapour can cause the carbon lattices to dissolve and produce many micropores. Compared to a one-step process, a two-step method yields more porous activated carbon generated from biochar. The biomass is first carbonized to biochar at a higher temperature, and the resulting biochar is then further activated by the activator to become activated carbon in two steps^34^. The activation mechanism is shown in the following equations.

$${\text{6KOH }} + {\text{ 2C }} \to {\text{ 2 K }} + {\text{ 3H}}_{{2}} + {\text{ 2K}}_{{2}} {\text{CO}}_{{3}}$$

$${\text{K}}_{{2}} {\text{CO}}_{{3}} + {\text{ C }} \to {\text{ K}}_{{2}} {\text{O }} + {\text{ CO}}_{{2}}$$

$${\text{K}}_{{2}} {\text{CO}}_{{3}} \to {\text{ K}}_{{2}} {\text{O }} + {\text{ CO}}_{{2}}$$

$${\text{K}}_{{2}} {\text{O }} + {\text{ C }} \to {\text{ 2 K }} + {\text{ CO}}$$

This work used pyrolysis at 500 °C to create granular-modified carbon using leftover sunflower stems as raw materials. The study's investigative objective is to modify, using KOH, the physicochemical characteristics of activated carbon and the way adsorbent species interact with adsorbates. The study aimed to evaluate the resultant KOH-activated sunflower stem-activated carbon (KOH-SSAC) utilizing FTIR, XRD, TEM, BET, SEM, and XPS methods.

The aqueous adsorption behaviour of KOH-SSAC was studied using model contaminants such as BPA and phenol. Ultimately, they examined the sorption properties, temperature, and time effects of the phenol and BPA, examining the influence of the activator, adsorption duration, and additional variables on the adsorption outcome. The high-value potential of biomass may be unlocked through the theoretical framework this study reveals.

## Materials and methods

### Materials

Potassium hydroxide (KOH), phenol, bisphenol (BPA), sodium hydroxide (NaOH), hydrochloric acid (HCl), and Acetonitrile (HPLC grade) were obtained from Samchun Pure Chemical Co. Ltd. (Korea). All the acquired reagents were of analytical grade and were used without the need for additional purification.

### Preparation of KOH-activated SSB

Sunflower stems are a type of agricultural debris that is imported from Gyeonggi-do, Seoul, South Korea. KOH-SSAC was made from sunflower stems using a two-step process that included pyrolysis and KOH activation. After the sunflower stem was crushed into a powder, any unwanted or floating material was washed away with distilled water. After that, it was heated at 65 °C for 24 h to dry it out. The dry products were pulverized, sieved, and then pyrolyzed for an hour at 500 °C under N_2_, with the temperature rising at a rate of 5 °C per minute. After quenching the resultant product with distilled water, it was again dried in an oven for a full day at 65 °C. This was also referred to as SSB (Sunflower Stem Biochar). After that, this SSB was activated using a 3:1 mass ratio of KOH and SSB. Subsequently, the resulting mixture was heated in a tube furnace using N_2_ at a rate of 5 °C per minute for one hour at 700 °C. The resulting adsorbent was named KOH-SSAC and utilized in additional investigations after being oven-dried and cleaned with 1 mol/L HCl.

### Material characterization

The X-ray powder diffraction patterns of the synthesized materials were acquired using a Rigaku D/Max-2500 diffractometer. Surface elemental composition was analyzed using the ESCALAB-210 X-ray photoelectron spectroscopy instrument (Spain). The SEM analysis of the sample was conducted using the Hitachi S-4300 and EDX-350 instruments from Japan. The Brunauer, Emmett, and Teller (BET) surface area and pore diameters of the materials were calculated based on data obtained from N_2_-physisorption measurements using an Autosorb-1 instrument from Quantachrome (USA). pH readings were obtained using a pH meter (DKK-TOA, HM-42X, Japan), and the pH of the solution was adjusted using 0.1 mol/L HCl and NaOH solutions. The residual radionuclide concentration was determined using an autosampler-fitted Optima 2100 DV inductive coupled plasma-optical emission spectroscopy (ICP–OES) instrument from PerkinElmer (USA).

### Adsorption experiments

The experiment aimed to investigate the adsorption behavior of KOH-SSAC on BPA and phenol in aqueous solutions. The range of the pollutants' concentration in the solutions, which was used to determine the adsorption capacity, was 10 to 500 mg/L. Batch adsorption studies were conducted using 20 mg of dried KOH-SSAC mixed with 50 mL of pollutant solution. Various adsorption periods were employed to collect the contact time and kinetic data. A phenol and BPA content was determined using HPLC equipment after the liquid samples were collected by filtering. A mobile phase containing methanol and water in a 50:50 ratio was utilized for phenol, whereas for BPA, a 40:60 mixture of water and ACN was used. A 20 µL sample was injected for phenol and BPA, and 0.4 ml and 0.60 mL of fluid, were flowed at different rates. The experiment was conducted three times, using three different pH values for the liquids. It was constantly agitated at 150 rpm while maintaining a steady temperature of 298 K. The adsorption capacity and kinetics of KOH-SSAC towards BPA and phenol from aqueous solutions were evaluated using these data. Optimizing the circumstances for the sorption of these pollutants from water resources is another application for the data. The comprehensive tests were carried out in triplicate, and the average outcomes were considered for additional computations. The equation used to obtain the adsorption capacity (q_e_) at equilibrium is shown in Eq. (1).

1 $${q}_{e}=\left({C}_{0}-{C}_{e}\right)* \frac{V}{m}$$

where q_e_ (mg/g) represents the pollutant's adsorption capacity at equilibrium, C_0_ (mg/L) is the starting concentration of the pollutant, Ce (mg/L) stands for the pollutant concentration at equilibrium, V (L) is the volume of the pollutant, and m (g) is the adsorbent weight.

## Results and discussion

### Characterization of activated carbon derived from biochar

**Figure 1:**
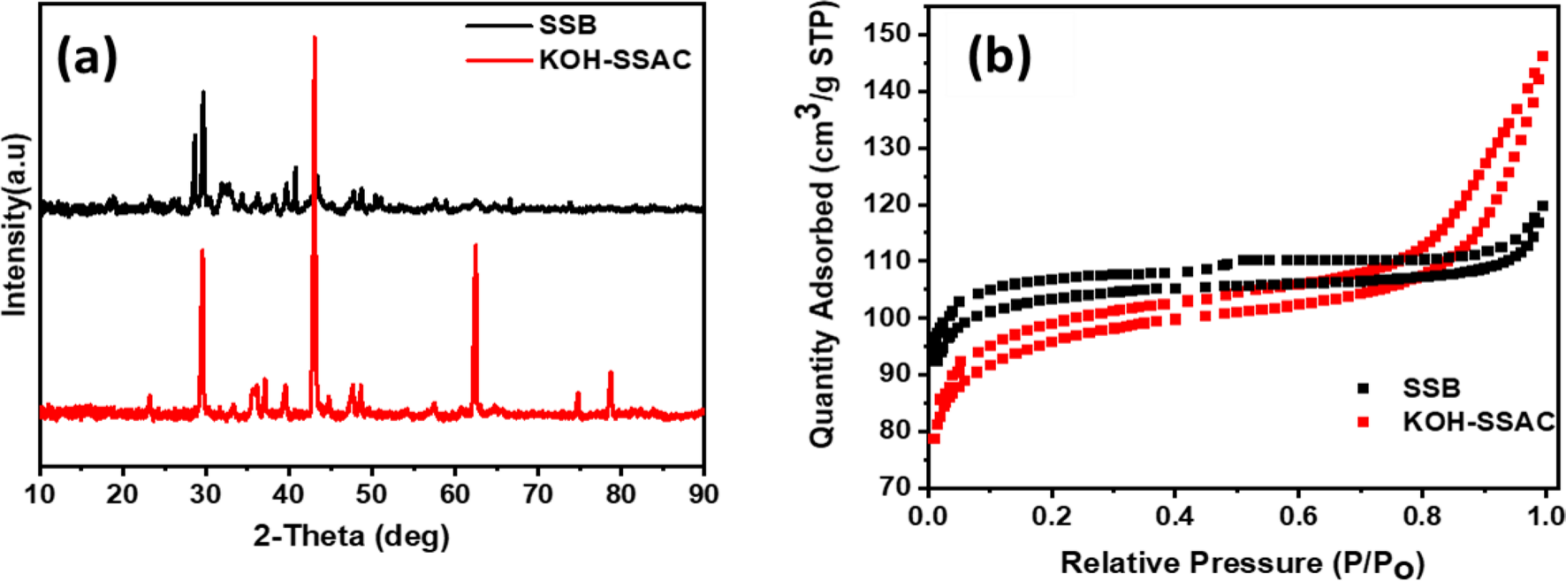
(**a**) XRD patterns of SSB and KOH-SSAC and (**b**) Nitrogen adsorption–desorption isotherms of SSB and KOH-SSAC.

Figure 1a illustrates the significant differences between the XRD analyses of the KOH-SSAC and SSB samples, highlighting the significance of the KOH activation process. Compared with SSB and KOH-SSAC, the match of two wide, distinctive peaks with centers at 23° and 44° is visible, as shown in Fig. 1a*.* These peaks correspond to graphite-aromatic and amorphous carbon structures' (0 0 2) and (1 0 1) diffractions, respectively^35^. In addition, KOH-SSAC exhibited several unique and sharp diffraction peaks. The loss of water and the cracking process cause the salting-out effect, which is responsible for the sharp diffraction peaks. This indicates that KOH-generated potassium salts were removed, leaving no trace impurities. However, they had a significant effect on the morphology of the SSB, causing it to hold on to more distinct crystalline forms, as evidenced by the peaks at nearly 61° and 77.5°, which correspond to crystalline carbon and graphite-like carbon nanofibers, respectively^36^. The BET analysis performed with the N_2_ desorption/adsorption isotherms of SSB and KOH-SSAC are depicted in Fig. 1b. The adsorption isotherms for both SSB and KOH-SSAC fall under the type IV isotherms as per the IUPAC classification, of both micro- and mesopore existence were confirmed. The BET surface areas were found to be 369.29 m^2^/g for SSB and 413.04 m^2^/g for KOH-SSAC. Evidently, after KOH activation, the specific surface areas (SBET) were increased, which is assumed to be responsible for the enhanced adsorption efficiency. Furthermore, pore volumes also decreased from 0.226 to 0.1853 cm^3^/g upon activation.

**Figure 2:**
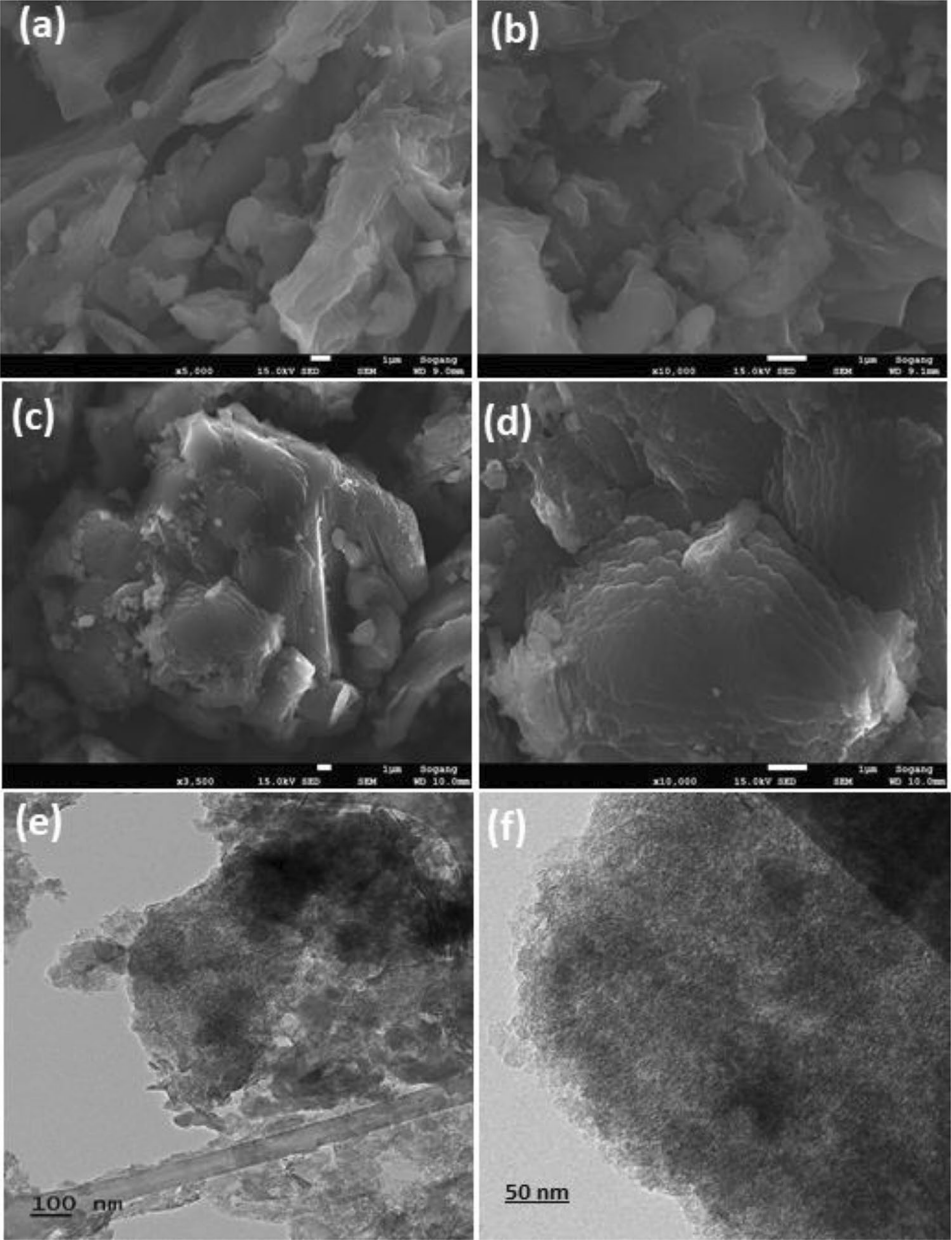
SEM images of (**a**, **b**) SSB, (**c**, **d**) KOH-SSAC, and (**e**, **f**) TEM images of KOH-SSAC.

FESEM microscopy and TEM are used to investigate surface morphology, including the textural aspects of prepared materials (Fig. 2). Figure 2a and b depict the surface of SSB, which exhibits a heterogeneous structure with irregular pores, lacking any definite shape. This results from the decomposition of hemicellulose and cellulose macromolecules^37^. However, after KOH activation, Fig. 2c and d demonstrate increased homogeneity of porosity with decreasing pore volume and a layered surface appearance in KOH-SSAC. SEM–EDX analysis of KOH-SSAC revealed elemental composition with an atomic percentage in the order of C > O > K > Ca > Cl > P. Notably, C and O exhibited higher concentrations compared to other elements, particularly with negligible concentrations observed for Cl and P, as illustrated in Fig. S1 and Table S1. The results of the TEM analysis provided valuable insights into the structural characteristics of the adsorbent made from sunflower stem biochar (SSB) after activation with KOH, as shown in Fig. 2e and f. The TEM analysis was conducted to investigate the origin of the mesoporous structure observed in KOH-SSAC. The sheet-like morphology observed in the TEM images suggests the formation of graphene-like layers within the KOH-SSAC material, which conforms to the previous results of FE-SEM and XRD. These layers contribute to developing a highly porous structure, providing ample surface area for adsorption observed in subsequent BET studies. The mesopores formed through the activation process play a crucial role in enhancing the adsorption capacity of the KOH-SSAC adsorbent. These results were found to be consistent with previous studies on KOH activation of biomass-derived materials and the resulting mesoporous structures^38,39^. The presence of sheet-like structures confirms the successful activation of SSB using KOH, leading to the development of a desirable mesoporous morphology.

### Effect of contact time and adsorption isotherms

**Figure 3:**
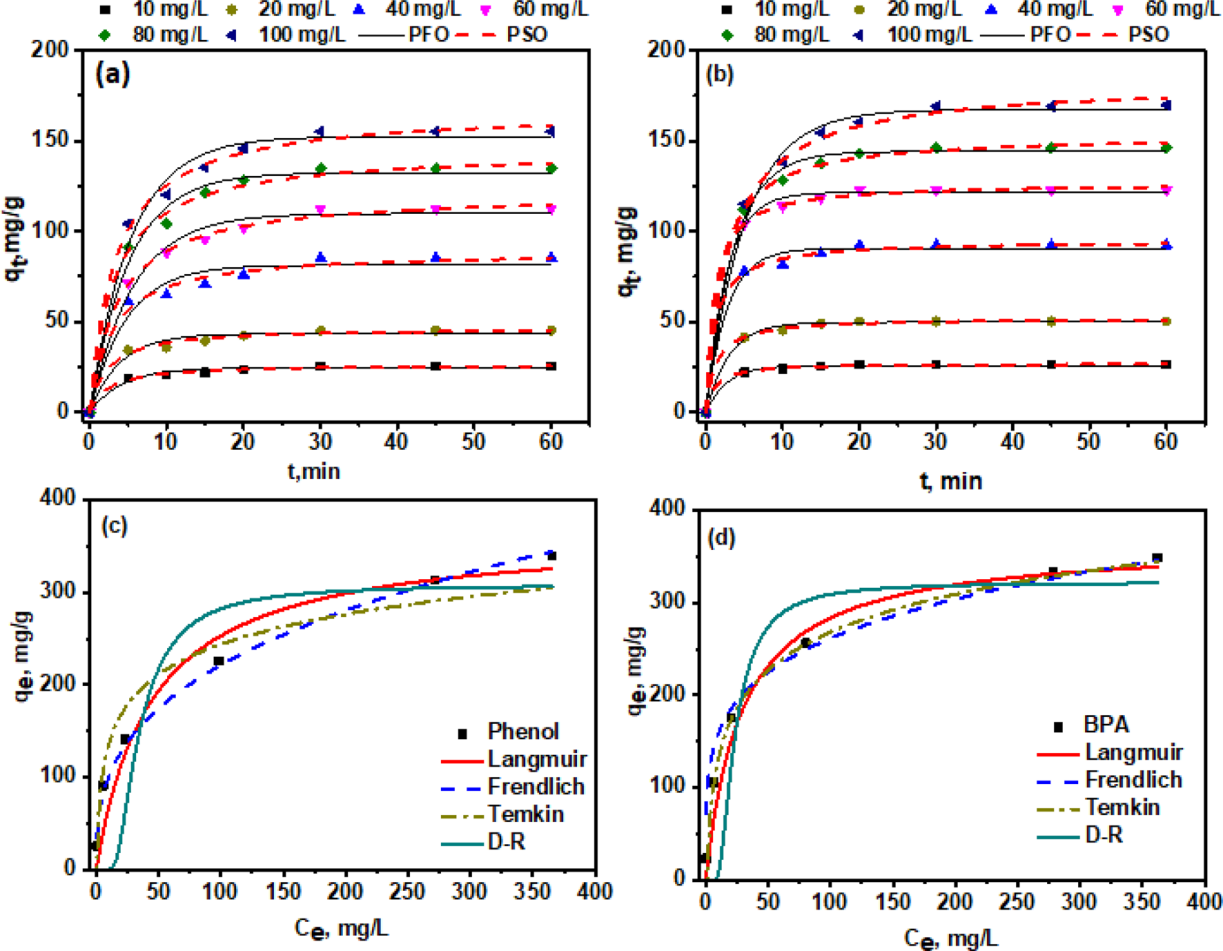
Kinetic effect (**a**, **b**) and Isotherms (**c**, **d**) of phenol and BPA at 0.4 g/L and pH of 6.

Investigations were carried out on the optimized adsorbent KOH-SSAC to comprehend the influence of contact period on the adsorption of BPA and phenol, with varying initial concentrations (10, 20, 40, 60, 80, and 100 mg/L). This is depicted in Fig. 3a and b. Notably, a significant portion of Phenol and BPA was adsorbed within 30 min, and then an incremental adsorption procedure was performed until equilibrium was reached. The swift initial adsorption rate results from the abundant adsorption sites on the KOH-SSAC surface, stemming from Van der Waals and electrostatic forces, as well as the quick diffusion of the pollutant toward these adsorbent surfaces. Conversely, the subsequent slower adsorption rate is attributed to the overabundance of active sites on the sorbent surface by adsorbates.

The study of adsorption kinetics is vital as it provides insightful information on the potential application of composites and a thorough knowledge of the adsorption process. Various models were introduced to elucidate the sorption mechanism. The obtained experimental data were fitted to the pseudo-first-order (PFO) and pseudo-second-order (PSO) models. These fitting outcomes were organized in Table 1, while the visual representation of Phenol and BPA removal on KOH-SSAC was demonstrated in Fig. 3a and b. This study critically evaluated the applicability of the PFO and PSO models in the context of Phenol and BPA adsorption onto KOH-SSAC adsorbent. These kinetic models facilitate the understanding of sorption mechanisms, shedding light on rate-controlling steps like transfer of mass and chemical reactions. Two kinetic models were employed to investigate these processes. The nonlinear kinetic models depicted the effect of time on the adsorption of both Phenol and BPA onto the developed KOH-SSAC. The calculated adsorption capacity (q_e_,_cal_) aligned well with the theoretical adsorption capacity (q_e,th_) derived from the PSO model. Notably, The PSO model's R^2^ value was much higher than the PFO model's, underscoring that the chemisorption of Phenol and BPA onto KOH-SSAC governs the rate-controlling stage of the removal process^40^. This indicates that chemical adsorption is the dominant factor, influenced by various factors such as electron-donor–acceptor interactions and hydrogen bonding.

**Table 1:** Phenol and BPA adsorption kinetics onto KOH-SSAC (0.4 g/L) at pH of 6.0 and 298 K ± 2.

Pollutants	Initial concentration, mg/L	*q*_*e th.*_, mg/g	Pseudo-first order ${q}_{t}={q}_{e}(1-{e}^{-k_{1}t})$			Pseudo-second order ${q}_{t} = \frac{{k}_{2}q_e^2t}{1+{k}_{2}{q}_{e}t}$		
			*q*_*e, Cal.*_, mg/g	*k*_*1*_	*R*^*2*^	*q*_*e, Cal.*_, mg/g	*k*_*2*_	*R*^*2*^
Phenol	10	25.58	24.49	0.239	0.970	26.5	0.015	0.992
	20	45.25	43.38	0.254	0.966	46.89	0.009	0.989
	40	85.35	81.43	0.211	0.953	89.20	0.003	0.991
	60	120.98	109.88	0.177	0.982	121.52	0.002	0.996
	80	143.65	132.43	0.194	0.984	144.86	0.002	0.994
	100	166.93	152.45	0.186	0.977	167.25	0.001	0.990
BPA	10	26.23	22.98	0.365	0.985	27.06	0.033	0.998
	20	50.28	47.76	0.334	0.984	52.20	0.014	0.997
	40	93.45	90.60	0.361	0.988	94.98	0.008	0.996
	60	123.56	121.56	0.371	0.986	126.72	0.007	0.998
	80	152.65	144.25	0.272	0.991	153.67	0.003	0.996
	100	179.65	167.38	0.193	0.981	182.76	0.0017	0.998

k_1_ (per min) and k_2_ (g/mg·min) are the rate constants; t: time (min).

Figure 3c and d illustrates the isotherms depicting the adsorption behaviour of phenol and BPA onto KOH-SSAC at 298 K under equilibrium conditions. To gain insights into the interactions between phenol, BPA, and the KOH-SSAC surface, various adsorption models, such as Langmuir, Freundlich, Temkin, and D–R, were employed to fit the experimental data. The corresponding model parameters and determination coefficients (R^2^) can be found in Table 2. The Freundlich model exhibited a significantly higher R^2^ value than the Langmuir, Temkin, and D–R models. Further, the statistical error and chi-square calculations (Table S2) found that Freundlich's model fitting shows a low chi-square value. These results suggest that each adsorption site on the KOH-SSAC surface possesses distinct adsorption energies and affinities for phenol and BPA^41^.

**Table 2:** Phenol and BPA adsorption isotherms onto KOH-SSAC (0.4 g/L) at pH 6.0 and 298 K ± 2.

Pollutants	Langmuir q_e_ = (q_max_K_L_Ce)/(1 + K_L_Ce)			Freundlich ${q}_{e}={K}_{F}+{{C}_{e}}^{1/n}$			Temkin ${q}_{e}=\frac{RT}{{B}_{T}}Ln{K}_{T}{C}_{e}$			D–R ${q}_{e}={q}_{D}-{\text{exp}}(\upbeta {\upvarepsilon }^{2})$		
	q_max_, mg/g	K_L_, L /mg	R^2^	K_F_, mg/g (L /mg)^1/n^	n	R^2^	B_T_	K_T_	R^2^	q_D_ (mg/g)	K_DR_ (mol^2^J^−2^) = $\upbeta {\upvarepsilon }^{2}$	R^2^
Phenol	333.03	0.012	0.896	46.04	2.94	0.992	46.92	1.91	0.888	308.39	3.11	0.760
BPA	365.81	0.034	0.924	96.68	4.62	0.986	59.11	0.93	0.724	322.09	24.98	0.622

q_max_ and q_D_: the maximum adsorption capacity; n: heterogeneity factor, K_L_, K_F_, K_T_ and K_DR_: constants of the Langmuir, Freundlich, Temkin, and D–R isotherms, respectively; B_T_: the heat of adsorption of in the Temkin.

Consequently, this leads to a non-uniform distribution of phenol and BPA on the surface, confirming the efficacy of multilayer adsorption for the adsorption of phenol and BPA by KOH-SSAC. From Langmuir isotherm, the maximum sorption capacity (q_m_) for phenol and BPA was determined to be 333.03 mg/g and 365.81 mg/g, respectively. KOH-SSAC shows high adsorption capacity for both pollutants when compared with SSB (258.15 mg/g for phenol and 289.25 mg/g for BPA) due to the KOH-SSAC having a high surface area. SSB isotherms are shown in Fig. S2 and Table S3 for comparison of results before and after activation of SSB (KOH-SSAC).

### Adsorption thermodynamic studies

**Figure 4:**
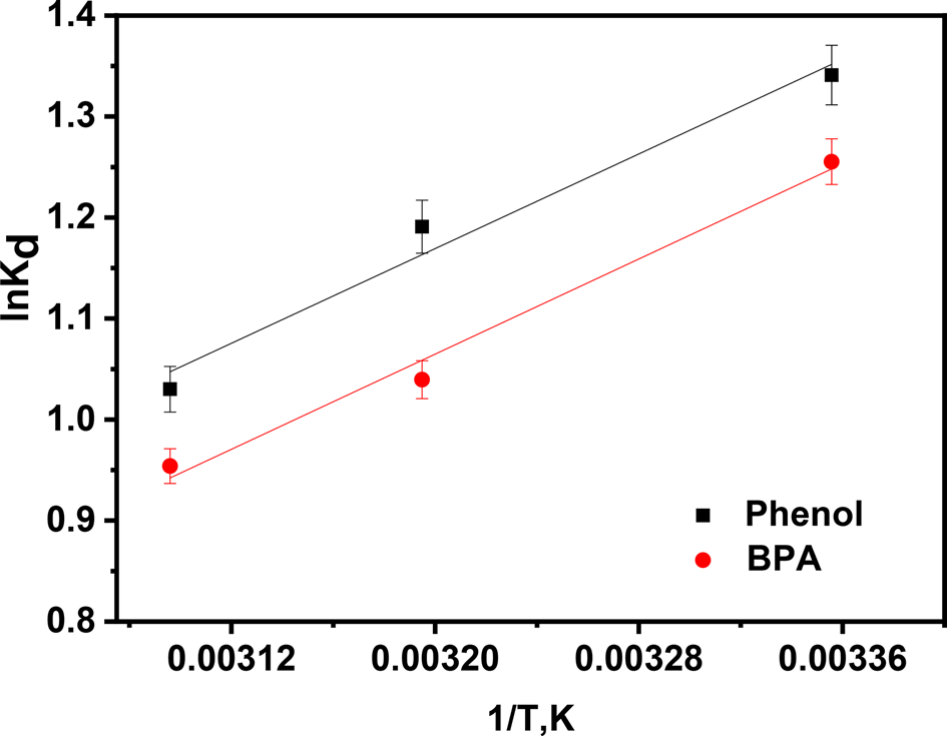
Effect of temperature on equilibrium constants for phenol & BPA (KOH-SSAC dosage = 0.4 g/L, and T = 298 K, pH 6.0, Time = 30 min).

The thermodynamics of phenol and BPA adsorption onto KOH-SSAC were analyzed under optimized conditions (pH 6, adsorbent dose = 0.4 g/L, contact time = 20 min, initial concentration = 100 mg/L) and across varying temperatures (298, 308, and 323 K) to assess the efficacy of the removal process. Thermodynamic parameters related to the adsorption, including ΔH (enthalpy, kJ/mol), ΔG (Gibbs free energy, kJ/mol), and ΔS (entropy, kJ/mol/K) as calculated using Eq. (2), played a pivotal role in predicting and characterizing the adsorption mechanism. Figure 4 illustrates a linear plot (lnK_d_ vs. 1/T) corresponding to the temperature-dependent equilibrium constant. The point of intersection on the Van't Hoff plots was employed to determine ΔH and ΔS.

2 $$\Delta G=-RTln{K}_{d}$$

K_d_ represents the thermodynamic equilibrium constant, C_q_ (mg/L) is adsorbed concentration of pollutant, T denotes the absolute temperature measured in Kelvin (K), and R is the ideal gas constant, with a value of 8.314 J/mol/K.

3 $${{\text K}_{\text d}=\frac{{\text C}_{\text q}}{{\text C}_{\text e}}}$$

4 $$ln{K}_{d}=\frac{\Delta s}{R}-\frac{\Delta H}{RT}$$

A diminution of the q_e_ for phenol and BPA with increasing temperature suggests an exothermic adsorption process. This phenomenon can be attributed to reducing the attractive forces involving the pollutant molecules and the active binding sites on the adsorbent surface^42^. Table 3 presents the results, indicating a negative sign for ΔG° at the studied temperatures. This implies that the sorption of phenol and BPA onto the adsorbent is thermodynamically favourable and occurs spontaneously. Moreover, these results underscore the effectiveness of KOH-SSAC in adsorbing phenol and BPA^43^. ΔG values falling between − 20 to 0 kJ/mol generally indicate physisorption processes^44^. The values of ΔG, along with their decreasing trend as temperature rises, further confirm that the adsorption of these compounds occurs primarily through physisorption. Similarly, the negative sign of the enthalpy change, ΔH°, signifies an exothermic adsorption behaviour. Additionally, the significant value of ΔS° indicates an increase in randomness at the thermodynamically stable interface throughout the adsorption process^43^.

**Table 3:** Thermodynamic parameters of the adsorption experiments (KOH-SSAC dosage = 0.4 g/L, and T = 298 K, pH 6.0, Time = 30 min).

Temperature, K	$\Delta G= -RT {\text{ln}}{K}_{d}$, (kJ/mol)	$\Delta H$, kJ/mol	$\Delta S$, kJ/mol K	lnKc Van't Hoff equation, ${\text{ln}}{K}_{d}=-\frac{\Delta H}{R\left(T\right)}+\frac{\Delta S}{R}$
Phenol				
298	− 1.341	− 9743.40	− 21.45	Y = 1171.9x − 2.5811, R^2^ = 9.756
313	− 1.190			
323	− 1.030			
BPA				
298	− 1.255	− 9793.06	− 22.48	Y = 1177.9x − 2.7047, R^2^ = 9.884
313	− 1.039			
323	− 0.953			

### Comparative study

Table 4 comprehensively compares the q_max_ of phenol and BPA across various adsorbents. In this study, we employed sunflower stem biochar treated with KOH as the adsorbent, and our findings revealed a commendable efficacy in the removal of both phenol and BPA. Compared to other adsorbents, KOH-SSAC consistently exhibited favourable results, showcasing its effectiveness as a potent adsorbent for the sorption of BPA and phenol from aqueous solutions.

**Table 4:** Comparison of phenol and BPA adsorption capacity on KOH-SSAC with reported adsorbents.

Adsorbent	Pollutant	q_max_ (mg/g)	pH	References
PbFe_2_O_4_ spinel-activated carbon	Phenol	145.71	7.0	^1^
Ortho-phosphoric acid-activated biochar		185.0	8.0	^16^
Sulphuric acid-activated biochar		154.39	8.0	^16^
Activated carbon		158.9	4.0	^17^
Soybean straw (SS) activated carbon (ZnCl_2_-SS)		278.0	~	^31^
Biochar-550 °C		26.73	6.5	^45^
KOH-SSAC		333.03	6.0	This study
Activated carbon fibers	BPA	277.78	7.0	^4^
Activated hydro chars		332.52	7.0	^6^
Goethite/activated carbon composite		187.21	7.0	^10^
Rice straw		181.82	2.35	^14^
Lignin-based activated carbon (KLP)		135.0	7.0	^40^
KOH-SSAC		365.81	6.0	This study

### Influence of external environmental conditions

**Figure 5:**
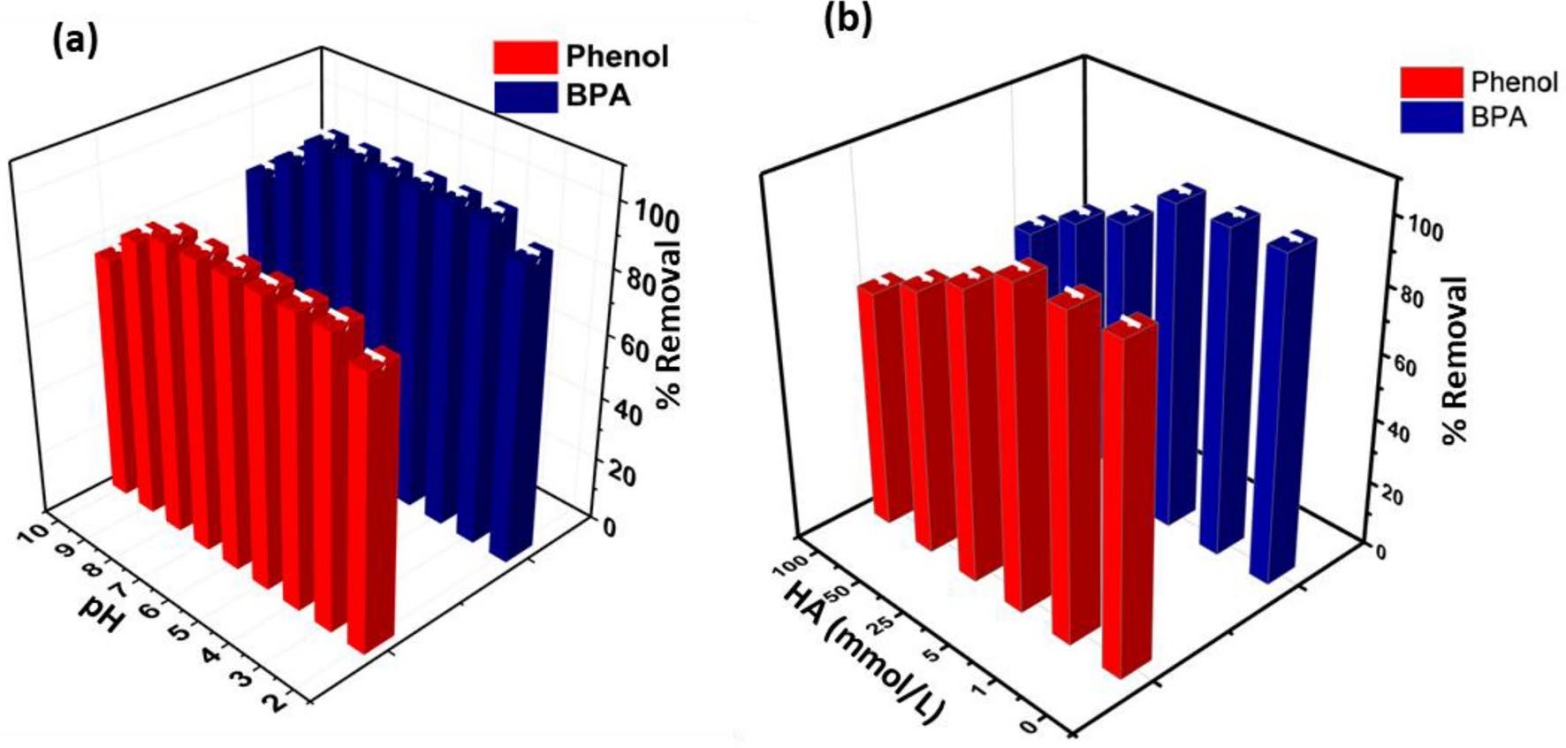
(**a**) pH Effect and (**b**) Humic acid influence on the removal of phenol & BPA on KOH-SSAC (KOH-SSAC dosage = 0.4 g/L, and T = 298 K, pH 6.0, Time = 30 min).

Among the various water quality parameters, ionic strength, pH, and organic matter significantly influenced sorption As depicted in Fig. 5a, pH had a notable impact on removing BPA and phenol. The effective sorption of these contaminants remained relatively stable across a pH range of 2 to 10. However, when the solution pH exceeded this range, the removal efficiency for phenol and BPA began to decline. This phenomenon can be ascribed to changes in the charge on the surface of KOH-SSAC and the nature of the pollutants. At higher pH values, the disassociation equilibrium of phenol led to the formation of its anionic form (C_6_H_5_O^−^) at pH > pKa (phenol, pKa = 9.92). This anionic form rendered the adsorption onto KOH-SSAC more challenging. Further reasons for the decline in adsorption efficiency include electrostatic repulsion between phenol anions and the KOH-SSAC surface and repulsion between phenolate-phenolate anions in the solution. Also, the stronger interactions between water and phenol anions caused by the higher solubility of phenol anions in aqueous solution made it harder for them to be displaced before adsorption^45^. When the pH was less than 9.0 (BPA, pKa = 9.6), the dominancy of BPA in the solution was the almost neutral molecule (BPA^0^). Hydrophobic interactions or hydrogen bonding between the contaminant and KOH-SSAC probably significantly eliminated BPA^46^. However, BPA molecules steadily lost protons as the pH of the solution rose over 9.5, transitioning in their ionic forms (BPA^−^ or BPA^2−^), leading to a rapid decrease in their hydrophobic interactions or hydrogen bonding with KOH-SSAC due to the increased hydrophilicity of BPA species^47^. Additionally, the functional groups of KOH-SSAC underwent partial deprotonation (pHzpc = 7.8), increasing the negative charge on KOH-SSAC. Consequently, electrostatic repulsion between KOH-SSAC and the protonated BPA species reduced the adsorption capacity under strong alkaline conditions.

**Figure 6:**
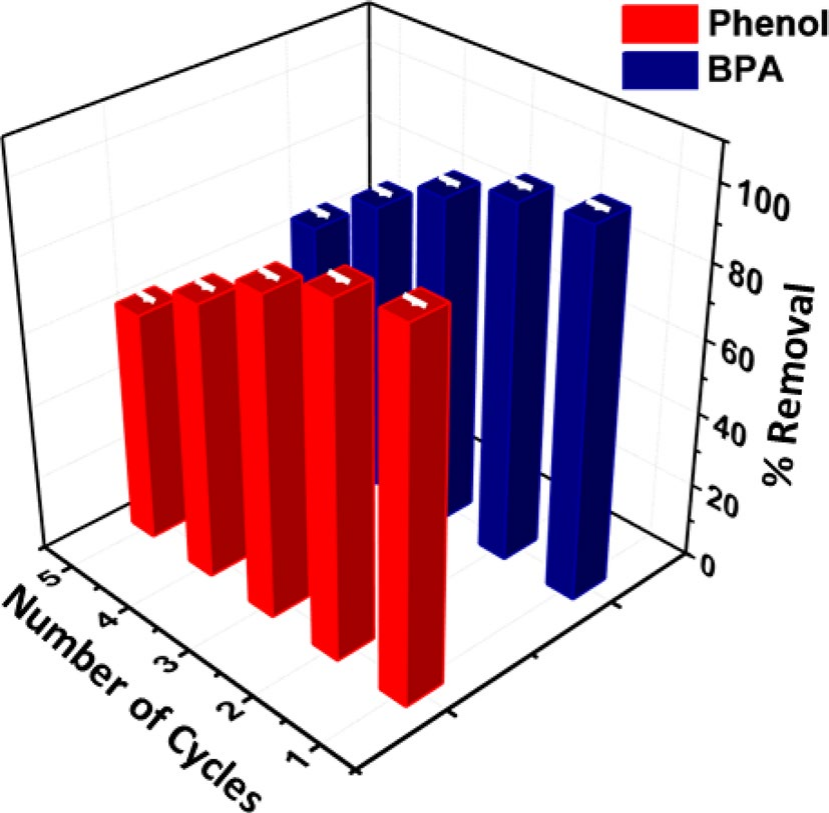
Regeneration studies on Phenol and BPA capture on KOH-SSAC (KOH-SSAC dosage = 0.4 g/L, and T = 298 K, pH 6.0, Time = 30 min).

Future investigated the impact of natural organic matter, specifically humic acid (HA), on removing phenol and BPA. As the concentration of HA increased, the removal efficiency of phenol and BPA by KOH-SSAC gradually declined, as illustrated in Fig. 6b. Notably, KOH-SSAC exhibited a robust resistance to HA interference at concentrations below 25 mg/L compared to the control sample. However, once the HA concentration reached 25 mg/L, its influence on the removal efficiency of both pollutants became negligible. This behaviour can be attributed to several factors. First, the competitive adsorption of HA led to a reduction in the available pore volume of KOH-SSAC through hydrophobic interactions or van der Waals forces. This reduction consequently resulted in a decline in phenol and BPA adsorption capacity^48^. Secondly, as HA was absorbed, it introduced oxygen-bearing surface functionality of the activated carbon surface. This addition had the effect of reducing the surface hydrophobicity of the activated carbon. As a result, the hydrophobic interactions between KOH-SSAC, phenol, and BPA were weakened, leading to decreased adsorption removal of these contaminants^49^.

### Desorption and regeneration studies

Adsorbents are sometimes discarded due to their inability to be regenerated, leading to their classification as secondary pollutants^50^. A regeneration process involving solvent treatment and heating was employed for the absorbent to address this issue. Subsequently, this regenerated adsorbent was used for the adsorption of BPA and phenol, with each adsorption experiment involving 20 mg of the regenerated adsorbent and 10 mg/L of phenol and BPA in a 50 mL solution. Like the adsorption tests, a 60-min desorption procedure was performed using a shaker. The process of separating the adsorbent from the liquid phase during adsorption and desorption was accomplished by centrifugation. The mixture underwent centrifugation at a speed of 4000 rpm for 10 min. After the desorption step, the adsorbent was meticulously cleansed at specific intervals using distilled water before oven drying. After the fifth cycle of heating and washing with 0.1 M NaOH, the regenerated adsorbent's maximum adsorption efficiency was measured, and there was a decrease in efficiency. Phenol decreased from 97.8 to 62%, while BPA dropped from 99 to 64%, as shown in Fig. 6. This decrease in effectiveness is caused by the interaction between phenol and NaOH, converting it into sodium phenoxide; it is water-soluble and aids in desorbing phenol from the adsorbent. The high regeneration ability of NaOH can be attributed to three simultaneous mechanisms: (1) The hydrolysis of oxygen groups on the adsorbent's surface and the interaction with the hydroxyl group (–OH) of phenol, (2) The significant solubility of phenol in water in alkaline conditions, (3) The repulsion between deprotonated acidic groups on the adsorbent's surface and the adsorbed phenol, induced by NaOH. A series of consecutive adsorption–desorption cycles were performed to evaluate the adsorbent's regeneration capability, totaling five cycles.

### Possible mechanism

Analysis of kinetics and isotherms revealed that the adsorption of phenol and BPA onto KOH-SSAC involved a mechanism combining physical and chemical properties. The PSO model suggested the presence of chemical interactions influencing the sorption process. Additionally, isotherm model fitting data revealed the formation of multilayer adsorption on KOH-SSAC. Interestingly, as the temperature increased, the adsorption capacity for phenol and BPA on KOH-SSAC decreased, indicative of an exothermic adsorption process. Moreover, higher initial concentrations of these contaminants facilitated their migration from the solution to the KOH-outer SSAC's surface. Notably, a larger pore volume within the adsorbent played a beneficial role in enhancing the removal process. The role of pore-filling, which is closely associated with the porous structure, was pivotal in the adsorption of contaminants on activated carbon. This resulted in elevated initial removal rates^51^.

**Figure 7:**
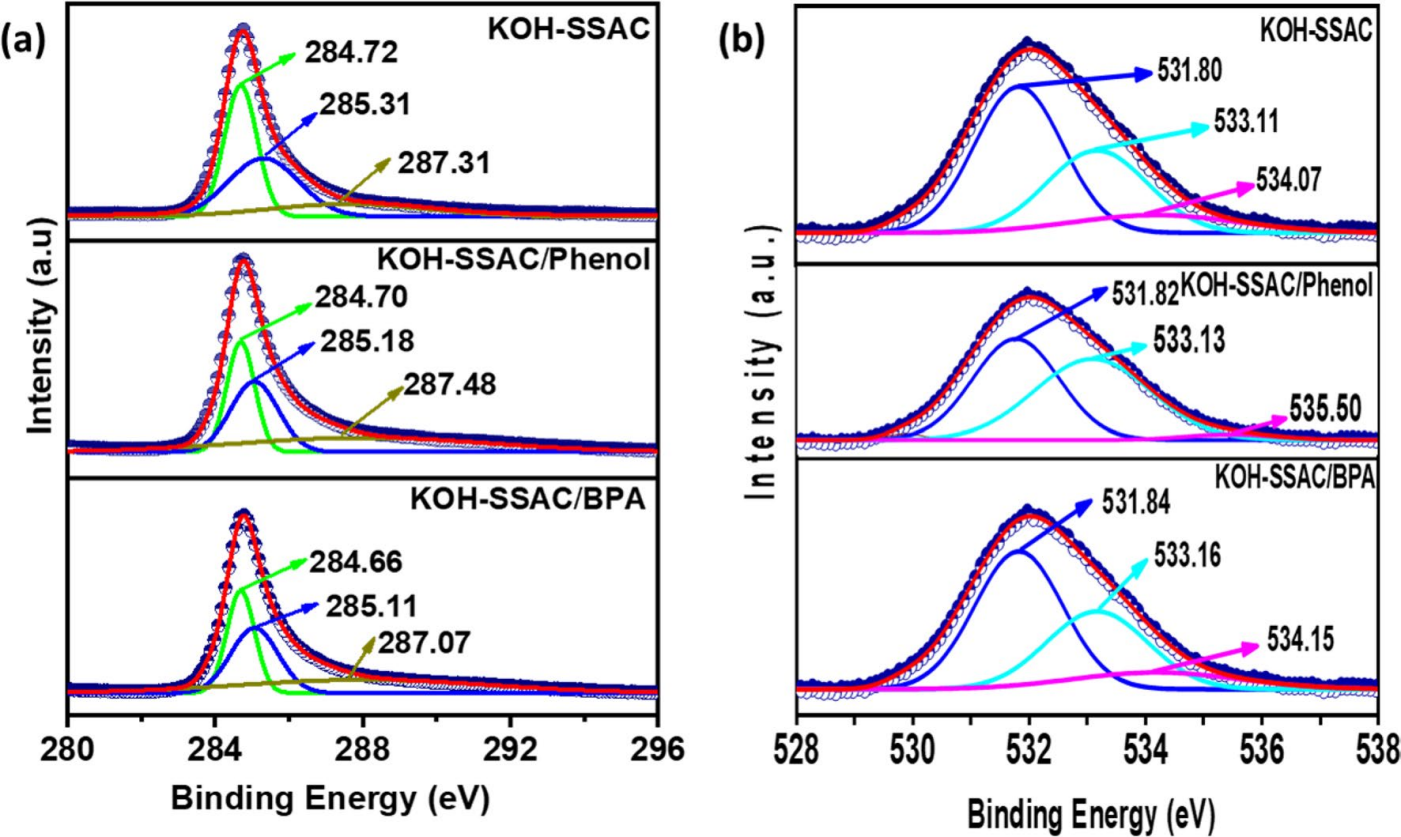
(**a**) C 1*s* and (**b**) O 1*s* XPS spectra of the region before and after the reaction.

Furthermore, π–π interactions represent a significant Mechanism of intermolecular interaction between the pollutants and KOH-SSAC. To gain insights into this adsorption mechanism, we conducted X-ray photoelectron spectroscopy (XPS) characterization on KOH-SSAC before and after the adsorption process, as portrayed in Fig. 7. In the C 1*s* spectra, the peaks at 284.72, 285.31, and 287.31 eV typically correspond to C–C/C=C, C–O, and C=O bonds, respectively^52^. Notably, after adsorption, these carbon functional groups shifted towards lower energy levels, which can be attributed to the adsorption of phenol and BPA. Particularly, certain groups, such as C–O, C–C, and C=O, are known to enhance their π-electron-donor potential towards BPA and phenol^53^. This suggests that π-π interactions are pivotal in facilitating the adsorption process.

**Figure 8:**
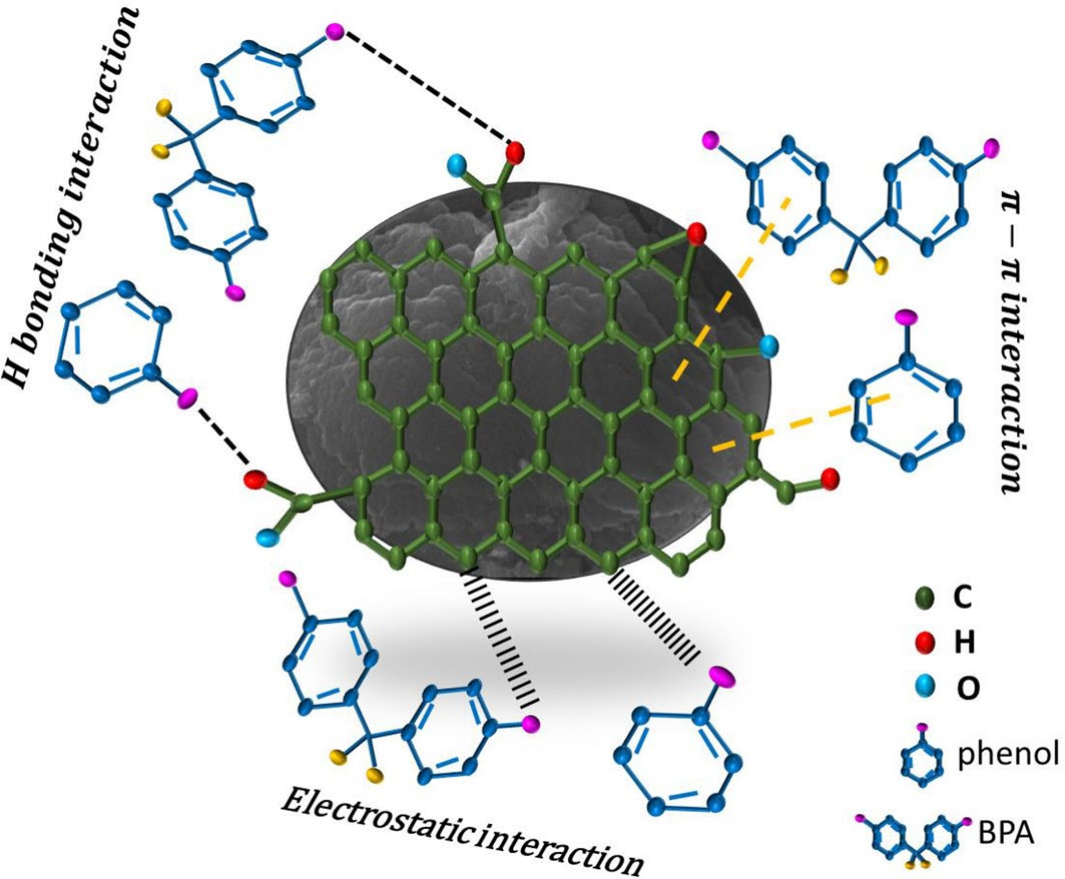
Mechanism of phenol and BPA adsorption on KOH-SSAC.

According to the hydrogen bonding process, surface functional groups on KOH-SSAC that include oxygen can establish hydrogen bonds with other functional groups, including the phenolic hydroxyl group found in organic compounds. This interaction promotes the sorption of pollutants onto the adsorbent. Simultaneously, water molecules can be adsorbed onto the activated carbon surface by forming hydrogen bonds. Subsequently, these water molecules act as secondary adsorption sites, forming clusters that prevent the penetration of organic substances into the activated carbon’s micropores^54^. Hydrogen bonds are the primary driving force behind the mobility of oxygen functional groups on the adsorbent's surface. This indicates that the oxygen-containing functionalities of phenol and BPA also participate in these interactions. In summary, Electrostatic interaction, π–π interactions, and hydrogen bonding substantially contribute to the adsorption capacity, and their mechanisms are illustrated in Fig. 8.

## Conclusions

In this research, KOH-activated carbon derived from sunflower stems (KOH-SSAC) exhibited remarkable efficacy as an adsorbent for phenol and BPA removal from aqueous solutions. The KOH activation process facilitated the generation of active surface sites and increased material porosity, significantly enhancing adsorption performance compared to SSB alone. Adsorption isotherm studies revealed impressive maximum adsorption capacities of 333.03 mg/g for phenol and 365.81 mg/g for BPA, surpassing other materials in similar studies. Thermodynamic analyses confirmed the spontaneous and exothermic nature of the adsorption process, with KOH-SSAC exhibiting robust regeneration capabilities and prolonged usage life. Mechanistic insights highlighted electrostatic attraction, π–π interactions, and hydrogen bonding as key contributors to its exceptional adsorption performance. Future directions include optimizing pore size distribution and surface area, exploring continuous column conditions, assessing synergistic treatment approaches, and conducting pilot-scale studies for practical water treatment applications. These efforts are essential for validating the effectiveness and feasibility of deploying KOH-SSAC on a larger scale, ensuring safe and efficient water treatment.

### Supplementary Information

Supplementary Information.

## Data Availability

The datasets are available from the corresponding author upon reasonable request.
