# Supplementary material for: Insights into kinetics, thermodynamics, and mechanisms of chemically activated sunflower stem biochar for removal of phenol and bisphenol-A from wastewater
Source: Sci Rep. 2024 Feb 21;14:4267. doi: 10.1038/s41598-024-54907-y (PMC10881974; doi:10.1038/s41598-024-54907-y)
Supplement: Supplementary file 1 — Supplementary Information. [file 41598_2024_54907_MOESM1_ESM.docx]

**Insights into kinetics, thermodynamics, and mechanisms of chemically activated sunflower stem biochar for removal of phenol and bisphenol-A from wastewater**

Lakshmi Prasanna Lingamdinne ^a^, Ganesh Kumar Reddy Angaru ^a^, Chandrika Ashwinikumar Pal ^a^, Janardhan Reddy Koduru ^a^*, Rama Rao Karri ^b^*, Nabisab Mujawar Mubarak ^b^, Yoon-Young Chang ^a^*

*^a^ Department of Environmental Engineering, Kwangwoon University, Seoul 01897, Republic of Korea*

*^b^Petroleum and Chemical Engineering, Faculty of Engineering, Universiti Teknologi Brunei, Bandar Seri Begawan, BE1410, Brunei Darussalam*

***Corresponding authors**. E-mail address: [reddyjchem@gmail.com](mailto:reddyjchem@gmail.com) (JR Koduru); [yychang@kw.ac.kr](mailto:yychang@kw.ac.kr) (YY Chang), [kramarao.iitd@gmail.com](mailto:kramarao.iitd@gmail.com) (RR Karri)


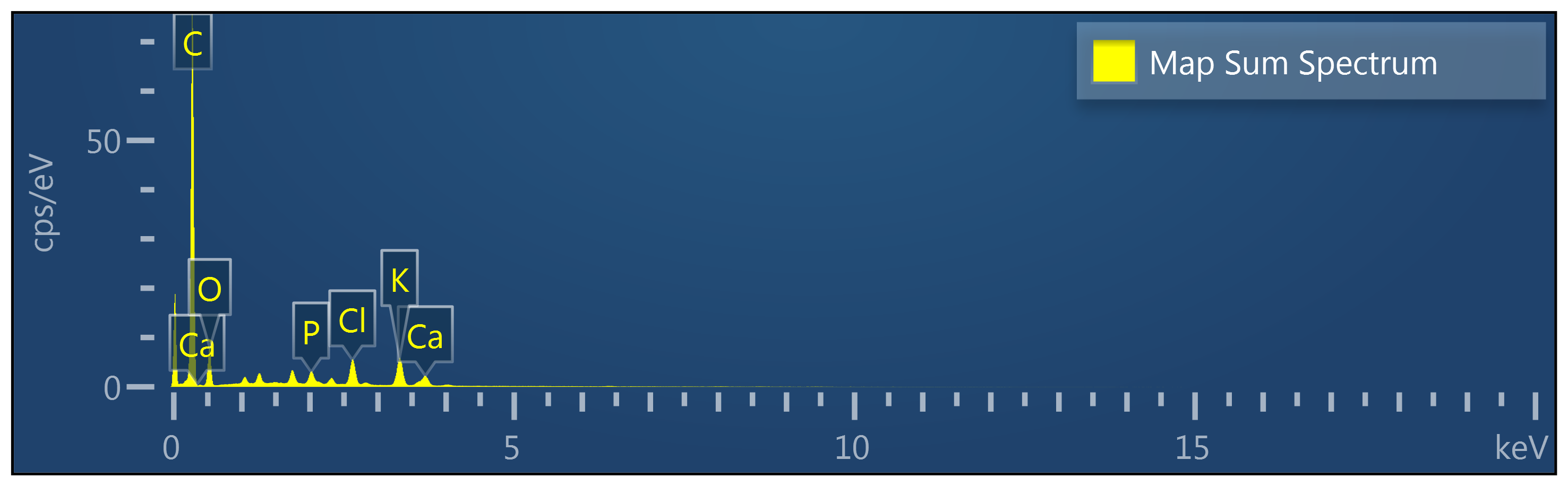


***Figure S1***: SEM-EDX of KOH-SSAC

***Table S1*:** Elemental composition of KOH-SSAC measured by SEM-EDX analysis.

| **Element** | **C** | **O** | **K** | **Ca** | **Cl** | **P** |
| --- | --- | --- | --- | --- | --- | --- |
| Atomic% | 86.50 | 11.30 | 0.92 | 0.36 | 0.67 | 0.25 |
| Weight% | 79.80 | 13.88 | 2.78 | 1.11 | 1.83 | 0.60 |

***Table S2*:** Statistical errors such as *chi-square* calculations to understand the suitability of isotherms for phenol and BPA on KOH-SSAC.

| **Pollutants** | **Langmuir** | **Freundlich** | **Temkin** | **D-R** |
| --- | --- | --- | --- | --- |
|  | χ^2^ | χ^2^ | χ^2^ | χ^2^ |
| phenol | 22.56 | 5.12 | 11.25 | 9.54 |
| BPA | 18.56 | 4.56 | 8.97 | 7.89 |


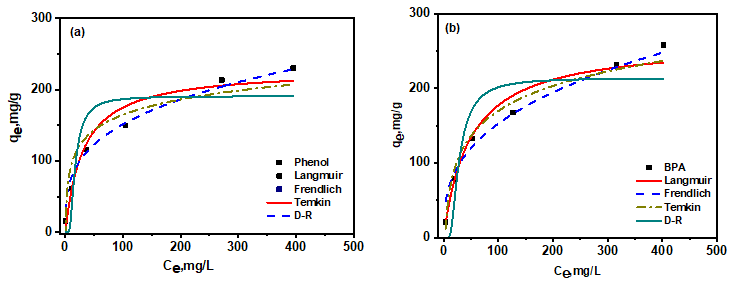


***Figure S2***: Isotherms (a& b) of phenol and BPA at 0.4g/L of SSB and pH of 6, and 298 ±2 K.

***Table S3*:** Phenol and BPA adsorption isotherms onto SSB (0.4g/L) at pH 6.0 and 298 ±2 K.

| **Pollutants** | **Langmuir** | | | **Freundlich** | | | **Temkin** | | | **D-R** | | |
| --- | --- | --- | --- | --- | --- | --- | --- | --- | --- | --- | --- | --- |
|  | q_max,_ mg/g | K_L_, L /mg | R^2^ | K_F_, mg/g (L /mg)^1/n^ | n | R^2^ | B_t_ | K_t_ | R^2^ | q_D_ (mg/ g) | K_DR_ (mol^2^J^−2^) | R^2^ |
| Phenol | 258.15 | 0.032 | 0.968 | 38.04 | 3.33 | 0.991 | 89.67 | 2.07 | 0.934 | 190.82 | 24.46 | 0.920 |
| BPA | 289.25 | 0.020 | 0.971 | 30.41 | 2.85 | 0.987 | 57.37 | 0.33 | 0.945 | 213.96 | 80.46 | 0.921 |
